# The Treatment of Tumours of the Bladder

**Published:** 1903-02-07

**Authors:** 


### THE TREATMENT OF TUMOURS OF THE BLADDER.

IN a lecture delivered at the Polyclinic on Tumours of the Bladder, Mr. P. J. Freyer[1] said that although hæmorrhage, pain, and other symptoms attendant on tumours of the bladder may be temporarily held in check by astringents and narcotics, the only line of treatment which offers a prospect of permanent relief consists in the complete removal of the growth. This used to be accomplished by means of perineal cystotomy, but this method has now for several years been practically abandoned in favour of operation through the supra-pubic route, which possesses the advantages that not only can a more thorough survey of the bladder be made in this way by the finger, but that the interior of that viscus and its contents can be brought into view, so that the tumour can be removed with greater precision than by the perineal route.

For the performance of the operation the Trendelenburg position advocated by some surgeons is quite unnecessary, as also is the use of Petersen's bag for inflating the rectum, which is attended by both inconvenience and danger. A rubber catheter is introduced, the urine is drawn off, and the bladder is thoroughly washed out with warm boric lotion, and is left distended with from 10 to 14 ounces of this fluid, the catheter being clipped and left in position.

The incision of from two to three inches in length is made in the middle line down to the muscles, terminating at the symphysis pubis. It is then continued either between the recti muscles or through one of them till the prevesical fat comes into view. The point of the forefinger being then passed down close behind the symphysis, the prevesical fat is scraped through by the finger-nail till the bladder is reached, and is drawn upwards off the bladder for the whole length of the wound, the peritoneum if extending low down being pushed up before it. The scalpel is then plunged through the bladder wall in the middle line with its edge directed downwards towards the symphysis, and an incision an inch or one and a-half inches long is made in this direction. If the patient be stout retractors may be necessary to separate the edges of the wound so as to bring the bladder into view, but it is not necessary to use a sharp hook, nor to insert sutures into the bladder to hold it up in position. The knife is now withdrawn and the finger introduced into the bladder plugging the opening, and then while the contained fluid is allowed to trickle away by the catheter the finger makes a methodical survey of the bladder.

The bladder being then again distended with lotion through the catheter (which is withdrawn till its end just reaches the inner orifice of the urethra, so as to be out of the way), the forceps is introduced by the side of the finger, and the growth, if pedunculated, is twisted off, and if sessile is removed by a process which consists partly of torsin and partly of biting it away. When the tumour has been removed as far as is possible in this manner, an ordinary glass Fergusson's vaginal speculum is introduced—the largest size that will readily pass through the incision in the bladder—and directed on the site of attachment of the growth, the lotion being allowed to flow away and the remainder being mopped up. By this means, and by aid of an electric forehead lamp, the site of the growth is brought clearly into view. Any shreds or nodules left can thus be removed, or the cautery can be applied, or tincture of perchloride of iron can be employed to stop hæmorrhage.

If necessary the tumour may be grasped boldly with the forceps and brought well up into the wound, a proceeding which will be facilitated by an assistant placing his finger in the rectum and pushing the base of the bladder up towards the suprapubic wound. The mucous membrane may then be incised all round the base of the growth, and this, together with the tumour, removed by serrated or cutting forceps. No attempt should be made to suture the wound in the mucous membrane.

The bladder is now thoroughly irrigated by warm boric lotion, through the catheter and out by the suprapubic wound, till all clots are removed and the fluid flows away clear. A wide drainage tube—so wide as to fill the wound in the bladder—is now introduced, and the abdominal wound is brought together round it by means of three or four silkworm-gut sutures, passed through skin and muscle, the drainage tube being fixed by a superficial suture. If the incision in the bladder should have been an extensive one it is to be partly closed by catgut before the tube is introduced. The end of the tube is cut about half an inch from the skin level, cyanised gauze, well wrung out of boric lotion, is applied, and the front and sides of the abdomen are covered with large pads of cotton or wood-wool, which dressings must be changed every three or four hours, as they become saturated with urine.

When a malignant growth is so extensive that no attempt at its removal is desirable or practicable, it may be necessary to open the bladder and establish permanent drainage, the perineal or suprapubic route being chosen, according to the position of the growth, so as to afford free drainage while avoiding pressure on the growth by the tube.

[1] Lancet, Jan. 24.

